# Current treatment landscape and translational priorities in malignant rhabdoid tumor of the kidney

**DOI:** 10.3389/fped.2026.1861712

**Published:** 2026-06-10

**Authors:** Zhigang Yao, Chenghao Zhanghuang, Nian Zhou, Jinrong Li, Zipeng Hao, Bing Yan, Hui Zhao

**Affiliations:** 1 Department of Urology, Kunming Children’s Hospital (Children’s Hospital Affiliated to Kunming Medical University), Kunming, China; 2 Urology Department, The First Affiliated Hospital of Kunming Medical University, Kunming, China; 3 Yunnan Province Clinical Research Center for Children’s Health and Disease, Yunnan Key Laboratory of Children’s Major Disease Research, Kunming, China; 4 Department of Dermatology, Kunming Children’s Hospital (Children’s Hospital Affiliated to Kunming Medical University), Kunming, China

**Keywords:** EZH2, malignant rhabdoid tumor of the kidney, pediatric renal tumor, precision oncology, SMARCB1, targeted therapy

## Abstract

Malignant rhabdoid tumor of the kidney (MRTK) is a rare and highly aggressive pediatric renal malignancy characterized by early dissemination, frequent presentation in infancy, and persistently poor survival despite multimodal therapy. Over the past decades, intensified treatment strategies combining surgery, multiagent chemotherapy, and selective radiotherapy have modestly improved outcomes, particularly in patients with localized disease. However, infants and those with stage III/IV or metastatic tumors continue to experience dismal prognosis, highlighting the limitations of further empiric escalation. The molecular hallmark of MRTK is loss of SMARCB1, a core subunit of the SWI/SNF chromatin-remodeling complex. This event provides a biologic framework for diagnosis, risk interpretation, and therapeutic development, and has shifted attention toward epigenetic and cell-cycle vulnerabilities. Early translational efforts, including EZH2 inhibition and CDK4/6 blockade, support proof of principle for biomarker-informed treatment, although renal-specific clinical evidence remains limited and single-agent activity has been modest. Immunotherapy and other emerging strategies are also being explored, but their roles in MRTK remain undefined because predictive biomarkers and disease-specific clinical datasets are lacking. In this Mini Review, we summarize the current treatment landscape of MRTK, examine why conventional multimodal therapy remains necessary but insufficient, discuss the preclinical model landscape that supports therapeutic translation, and define major priorities required to move the field toward renal-specific precision care. We argue that the next meaningful advance will depend on collaborative trials that integrate molecular profiling, faithful experimental models, rational combinations, and correlative biomarker studies from diagnosis onward.

## 1 Introduction

Malignant rhabdoid tumor of the kidney (MRTK) is one of the most aggressive renal malignancies in early childhood. Although it accounts for only a small proportion of pediatric renal tumors, its clinical significance is disproportionate because of its rapid progression, frequent metastatic spread, and poor survival compared with Wilms tumor and other more common childhood kidney neoplasms (1–3). MRTK typically presents in infancy or very early childhood, and unfavorable outcomes are particularly common in patients younger than 1 year, in those with advanced local disease, and in those with distant metastases at diagnosis (4–10).

The management of MRTK has historically relied on multimodal therapy, including surgery, intensive chemotherapy, and selected radiotherapy. Cooperative-group studies suggest that treatment intensification has improved outcomes to some extent, especially in patients with localized disease; however, survival gains in advanced-stage or metastatic disease have remained limited (5–7). This persistent therapeutic plateau has raised an important clinical question: whether further empiric escalation of cytotoxic therapy is likely to provide meaningful benefit, or whether future progress will depend more on molecularly informed treatment strategies.

The defining molecular feature of MRTK is loss of SMARCB1, also known as INI1 or BAF47, a core subunit of the SWI/SNF chromatin-remodeling complex (11–15). This alteration has transformed the conceptual framework of the disease. MRTK is now better understood as a SMARCB1-deficient epigenetic malignancy with characteristic vulnerabilities in chromatin regulation, cell-cycle control, and stress-response pathways. At the same time, modern pediatric precision oncology initiatives increasingly demonstrate that integrated molecular profiling can refine diagnosis, reclassify risk, identify germline predisposition, and guide matched therapy or trial allocation even when a single immediately actionable mutation is absent (16–21). These broader developments make MRTK an instructive model disease for molecularly guided pediatric cancer care. In this Mini Review, we summarize the current treatment landscape of MRTK, discuss why conventional multimodal therapy remains necessary but insufficient, evaluate the emerging preclinical model landscape, and highlight the translational priorities that may enable more effective renal-specific precision care.

## 2 What conventional multimodal therapy has achieved—and where it still fails

Complete surgical resection remains a central component of MRTK management whenever technically feasible. Nephrectomy provides histopathologic confirmation, contributes to local disease control, and supplies staging information that directly influences subsequent therapy (1, 4–6). However, surgery alone is rarely curative because many patients present with biologically aggressive disease and early occult dissemination. For this reason, multimodal treatment incorporating surgery, multiagent chemotherapy, and selected radiotherapy has become the foundation of current care (5, 6, 13, 22).

Over time, intensified treatment regimens have produced some measurable progress, but these gains have been uneven. Comparative analyses from cooperative-group studies, including the transition from NWTS-5 to AREN0321-based approaches, suggest that more intensive therapy can improve outcomes overall, yet the benefit appears to be concentrated largely in patients with stage I/II disease (6). By contrast, children with stage III/IV tumors, metastatic presentation, or very young age at diagnosis continue to have poor survival despite aggressive treatment. This distinction is clinically important because it indicates that the main limitation of current therapy is not failure to control all localized disease, but inability to overcome the biologic aggressiveness of high-risk MRTK.

The role of radiotherapy remains clinically relevant but complex. Population-based and registry-derived studies suggest that radiotherapy may contribute to improved local control and possibly better survival in selected patients, especially after surgery and chemotherapy in localized disease settings (9, 10). Nevertheless, the use of radiotherapy in infants and very young children must be weighed against substantial long-term toxicity, including growth disturbance and late organ effects. Proton therapy is conceptually attractive because it may reduce radiation exposure to surrounding normal tissues, but renal-specific evidence remains sparse and is still based mainly on case-level or limited retrospective experience rather than prospective comparative trials (11).

Similarly, the value of high-dose chemotherapy with stem cell rescue remains uncertain. Available retrospective studies have not consistently shown a clear survival advantage, while treatment-related toxicity is substantial (8). Taken together, these data support a practical conclusion: conventional multimodal therapy remains indispensable in MRTK because it provides the only established framework for initial disease control, but further universal intensification is unlikely to transform outcomes across all risk groups. The central unmet need is therefore not simply more therapy, but more effective biologic stratification and mechanism-based treatment integration.

## 3 SMARCB1 loss as the biologic framework for treatment development

The loss of SMARCB1 is the molecular hallmark of MRTK and the key biologic event that organizes both diagnosis and therapeutic reasoning. As a core component of the SWI/SNF chromatin-remodeling complex, SMARCB1 plays an essential role in ATP-dependent chromatin accessibility, transcriptional regulation, and maintenance of differentiation programs (12–14). Its absence disrupts normal enhancer function, favors transcriptional repression, and creates a tumor state that depends less on numerous kinase mutations and more on epigenetic dysregulation. This helps explain why MRTK typically shows a relatively simple genomic landscape but remains clinically devastating.

From a pathology-oriented perspective, loss of nuclear SMARCB1/INI1 expression is a key diagnostic anchor for MRTK, particularly in small biopsies, metastatic lesions, or tumors with overlapping rhabdoid morphology. However, INI1 loss should not be interpreted in isolation. It must be integrated with age, renal site, morphology, immunophenotype, and molecular findings, because the differential diagnosis includes Wilms tumor with rhabdoid features, clear cell sarcoma of the kidney, congenital mesoblastic nephroma, renal medullary carcinoma, epithelioid sarcoma, and other SWI/SNF-deficient neoplasms of the genitourinary tract. This diagnostic framework is clinically important because accurate recognition of SMARCB1-deficient MRTK directly influences risk stratification, genetic counseling, and eligibility for biomarker-driven trials (23, 24).

From a treatment perspective, this biology is highly informative. It suggests that the limitations of conventional chemotherapy are not merely related to insufficient dose intensity, but also to the fact that cytotoxic therapy does not directly address the transcriptional and epigenetic programs established by SWI/SNF disruption. At the same time, the biology of MRTK also argues against a simplistic replacement model in which targeted therapy would immediately substitute for surgery or chemotherapy. Most children with newly diagnosed MRTK still require urgent local and systemic disease control, and mechanism-based therapies are currently more realistic as complementary or combination strategies than as frontline replacements.

SMARCB1 loss also has implications beyond drug development. A subset of patients carry germline pathogenic variants associated with rhabdoid tumor predisposition syndrome, which influences family counseling, surveillance planning, and interpretation of disease risk (15). Recent clinical genomics studies in childhood cancer further reinforce the value of integrated profiling for uncovering diagnostically and therapeutically relevant somatic and germline findings beyond conventional histopathology alone (23–33). Accordingly, molecular assessment should not be viewed as an optional test reserved for relapse or research settings. Early confirmation of SMARCB1 deficiency, careful pathologic evaluation, and consideration of germline testing in appropriate clinical contexts are increasingly relevant to modern MRTK management.

## 4 Emerging targeted and immune-based strategies: promise, limits, and evidence gaps

Among targeted approaches in SMARCB1-deficient tumors, EZH2 inhibition is currently the most clinically advanced. Because loss of SMARCB1 function shifts chromatin regulation toward PRC2-mediated repression, EZH2 represents a biologically plausible dependency target (12–14). The pediatric MATCH experience with tazemetostat supports proof of principle that biomarker-informed epigenetic therapy can alter disease behavior in selected SMARCB1- or SMARCA4-altered tumors (16). However, the clinical meaning of these data for MRTK requires careful interpretation. The main value of tazemetostat to date is not evidence of strong single-agent efficacy in renal rhabdoid tumors, but confirmation that epigenetic targeting in this molecular context is feasible and biologically relevant. For MRTK specifically, renal-focused efficacy data remain limited.

CDK4/6 inhibition represents a second rational strategy. SMARCB1 loss has been linked to dysregulation of the cyclin D-CDK4/6-RB axis, which provides a mechanistic rationale for cell-cycle targeting (12, 14). Pediatric early-phase trials of ribociclib have demonstrated feasibility in malignant rhabdoid tumors and other solid tumors, yet objective responses have been limited and clinically useful predictive biomarkers remain immature (17). As a result, the field has gradually shifted from evaluating CDK4/6 inhibitors as standalone interventions toward exploring them as components of rational combination regimens, for example with epigenetic agents or therapies designed to increase apoptotic vulnerability.

A more critical interpretation of targeted therapy is therefore required. Resistance to mechanism-based agents in MRTK is likely to be multifactorial rather than attributable to a single escape pathway. Potential contributors include incomplete suppression of epigenetic dependency, adaptive enhancer reprogramming, compensatory cell-cycle bypass, persistence of drug-tolerant cell states, inadequate apoptotic priming, and pharmacologic constraints related to dose intensity and toxicity in infants. These considerations help explain why biologically rational agents such as EZH2 inhibitors or CDK4/6 inhibitors may produce disease stabilization without durable tumor eradication. Future studies should therefore prioritize rational combinations, serial molecular assessment, and model-linked resistance analyses rather than relying solely on single-agent response rates (12, 14, 16, 17, 32, 34).

Immunotherapy remains an area of interest, but its role in MRTK is currently undefined and should be interpreted with particular caution in infant disease. Studies across SMARCB1-deficient malignancies suggest that some tumors may display immune-cell infiltration, checkpoint-related signaling, or occasional durable responses to immune-based therapy (18). However, these observations cannot be directly generalized to MRTK. Infant MRTK may have a distinct host immune context, low mutational burden, rapidly progressive clinical behavior, and treatment-related immune suppression caused by intensive chemotherapy, all of which may reduce the likelihood of predictable benefit from immune checkpoint blockade alone. In addition, renal-specific MRTK cohorts with integrated PD-L1 expression, tumor-infiltrating lymphocyte profiling, antigen-presentation markers, myeloid-cell composition, and spatial immune architecture are still lacking. Consequently, immunotherapy should currently be regarded as an investigational strategy that requires biomarker-rich trials, combination approaches, and careful age-specific toxicity evaluation rather than as an established therapeutic option.

Other emerging strategies are promising but remain preclinical. Bioinformatic analyses have also identified mTORC1-related signaling signatures associated with prognosis in MRTK, suggesting additional opportunities for biomarker development and pathway-directed intervention (20). These include exportin-1 inhibition, FOXM1 targeting, and ferroptosis-related approaches involving pathways such as YTHDF1/GSTM2 signaling (19–21). In parallel, a 2026 international pediatric therapeutic development workshop on rhabdoid tumors emphasized priority targets and combinations such as DCAF5, MDM2, selective inhibitors of nuclear export, and next-generation EZH2-directed approaches, but also highlighted the need for more rigorous preclinical selection and better aligned early-phase development across renal and extra-renal rhabdoid tumors (25). Collectively, these findings broaden the field beyond classical epigenetic therapy and suggest that transcriptional state, nuclear transport, and metabolic stress responses may represent exploitable vulnerabilities in rhabdoid biology. Nonetheless, none of these avenues has yet generated sufficient renal-specific clinical evidence to influence current frontline practice. This evidence gap makes disease-relevant experimental models central to the next stage of MRTK therapeutic development.

## 5 Translational priorities for renal-specific precision care

A major challenge in MRTK research is that much of the available evidence is extrapolated from broader categories such as extracranial rhabdoid tumors, pooled SMARCB1-deficient cancers, or mixed pediatric solid tumor trials. Although these datasets are useful for identifying biologic principles and candidate drugs, they do not fully resolve the clinical questions that matter most for renal-specific decision-making. MRTK differs from extra-renal rhabdoid tumors in anatomic context, local treatment demands, age distribution, and patterns of presentation, all of which may influence therapeutic timing, tolerability, and outcome interpretation. As a result, reliance on pooled rhabdoid data can obscure the specific translational needs of children with primary renal disease.

The first priority is earlier and more systematic molecular integration at diagnosis. Recent pediatric precision oncology programs consistently show that comprehensive DNA/RNA profiling can improve diagnosis, reveal cancer predisposition, support risk refinement, and increase access to matched therapies or trials in high-risk pediatric cancers (26–33). In MRTK, such integration should include prompt confirmation of SMARCB1 status, consideration of germline evaluation where appropriate, and multidisciplinary interpretation that links pathology with molecular findings from the outset of care.

The second priority is the development of renal-specific collaborative trial designs. Given the rarity of MRTK, single-center or even single-group experiences will remain underpowered. However, future trials should prospectively preserve renal subgroup analyses rather than subsuming MRTK entirely within pan-rhabdoid or pan-SMARCB1 cohorts. Master-protocol approaches, including basket, umbrella, and platform frameworks, may improve efficiency in rare biomarker-defined cancers, but for MRTK these designs should still preserve clinically meaningful renal-context analyses and correlative endpoints (25, 29, 35).

The third priority is incorporation of correlative science into clinical studies. The field does not yet know which patients are most likely to benefit from EZH2 inhibition, cell-cycle blockade, immune-based therapy, or future combinations. Longitudinal biospecimen collection, patient-derived models, organoid platforms, and treatment-linked biomarker studies will be essential to identify mechanisms of response, resistance, and disease evolution. Recent organoid-based work in malignant rhabdoid tumors has already demonstrated tractable metabolic vulnerabilities, supporting the translational value of model systems that are biologically closer to patient tumors than standard cell lines alone (32). In a rare tumor such as MRTK, this translational infrastructure is not optional; it is necessary if clinical trials are to generate more than descriptive efficacy signals.

The fourth priority is model-linked translational infrastructure. Molecular profiling should be paired with systematic efforts to generate patient-derived organoids, PDXs, and orthotopic or metastatic models from the same clinical cases whenever feasible. Linking these models to clinical annotation and longitudinal biospecimens would allow candidate vulnerabilities to be tested across complementary platforms and would reduce the risk that therapeutic decisions are based on model-specific artifacts rather than reproducible MRTK biology.

A closely related priority is the establishment of faithful preclinical platforms that can convert molecular findings into experimentally testable therapeutic strategies; this issue is discussed in detail below.

## 6 Preclinical models and translational research platforms in MRTK

A focused assessment of the preclinical landscape is essential because the therapeutic implications of SMARCB1 loss cannot be converted into testable strategies without disease-relevant models. For MRTK, available resources can be grouped into established cell lines, xenograft and patient-derived xenograft (PDX) platforms, patient-derived organoids, and genetically engineered or SMARCB1-reconstitution systems. Each platform addresses a different question, but none fully captures renal origin, infant biology, metastatic dissemination, and treatment response simultaneously.

Conventional two-dimensional cell lines remain useful for rapid mechanistic work, genetic perturbation, and early drug screening. G-401 is a particularly instructive example: although it was historically used in some Wilms tumor studies, subsequent pathologic and molecular reassessment showed that it derives from a rhabdoid tumor of the kidney and harbors the characteristic SMARCB1 abnormality (36). This gives G-401 relevance for SMARCB1-deficient biology, but long-term culture adaptation, absence of immune and stromal components, and limited capacity to model renal microenvironment or metastatic evolution constrain direct clinical extrapolation. Therefore, findings from cell-line studies should ideally be validated in orthogonal *in vivo* or organoid-based systems.

Xenograft and PDX models add an *in vivo* context that is particularly important for assessing tumor growth, stemness, invasion, and pharmacologic response. Human MRT has been propagated *in vivo* as serial xenografts, enabling analysis of cancer stemness, invasion and motility signatures, and target discovery (37). In pediatric renal tumor studies, preclinical models have also supported evaluation of vulnerabilities such as XPO1 inhibition (19), and organoid-based drug-screening findings such as neddylation inhibition have been extended to an MRT PDX model with survival benefit *in vivo* (38). However, PDX platforms remain slow, costly, and low throughput; they are usually maintained in immunodeficient hosts; and subcutaneous implantation may not reproduce renal-site biology or metastatic spread. These limitations are particularly relevant for MRTK because renal origin, young age, and early dissemination are central features of the clinical disease.

Patient-derived organoids represent the most notable recent advance in pediatric renal tumor modeling. A pediatric kidney tumor organoid biobank included Wilms tumors, MRTK, renal cell carcinomas, and congenital mesoblastic nephromas; MRTK organoids were generated from all available MRTK samples and retained disease identity at phenotypic, genomic, transcriptomic, and epigenetic levels (39). Subsequent MRT organoid studies showed that organoids can identify selective vulnerabilities such as neddylation and nucleotide synthesis, while patient-derived organoids have also enabled SMARCB1 reconstitution and multi-omics analysis of enhancer reprogramming (32, 34, 38). These platforms are scalable and compatible with drug screening, but they still incompletely represent vasculature, immune contexture, stromal interactions, systemic drug exposure, and metastatic niche selection.

Comparison with Wilms tumor is instructive, not only from the perspective of model development but also from the perspective of tumor microenvironment research. Wilms tumor research has historically been constrained by scarce, heterogeneous, and sometimes misclassified models; nevertheless, recent work has produced a broader spectrum of platforms, including patient-derived xenografts, organoid biobanks, primary cultures, CRISPR-edited organoids, PDX-derived organoids, and partially reprogrammed or induced pluripotent stem cell-like systems that capture high-risk blastemal features and metastatic behavior (39, 40). In parallel, recent Wilms tumor studies have increasingly emphasized immune evasion, stromal-immune interactions, natural killer-cell biology, macrophage polarization, and subgroup-specific immune contexture as important determinants of future immunotherapeutic strategies (41). This comparison is relevant to MRTK because it illustrates how translational progress in pediatric renal tumors depends on biologically faithful models that preserve not only tumor-intrinsic programs but also microenvironmental and immune features. MRTK has not yet achieved a comparable diversity of renal-specific platforms. Although MRTK organoids and PDXs represent meaningful progress, available models remain relatively few and are often embedded within broader MRT or SMARCB1-deficient datasets. This scarcity limits reproducible comparison of drug sensitivity by renal origin, age, stage, metastatic phenotype, immune contexture, and prior treatment exposure.

**Figure 1:**
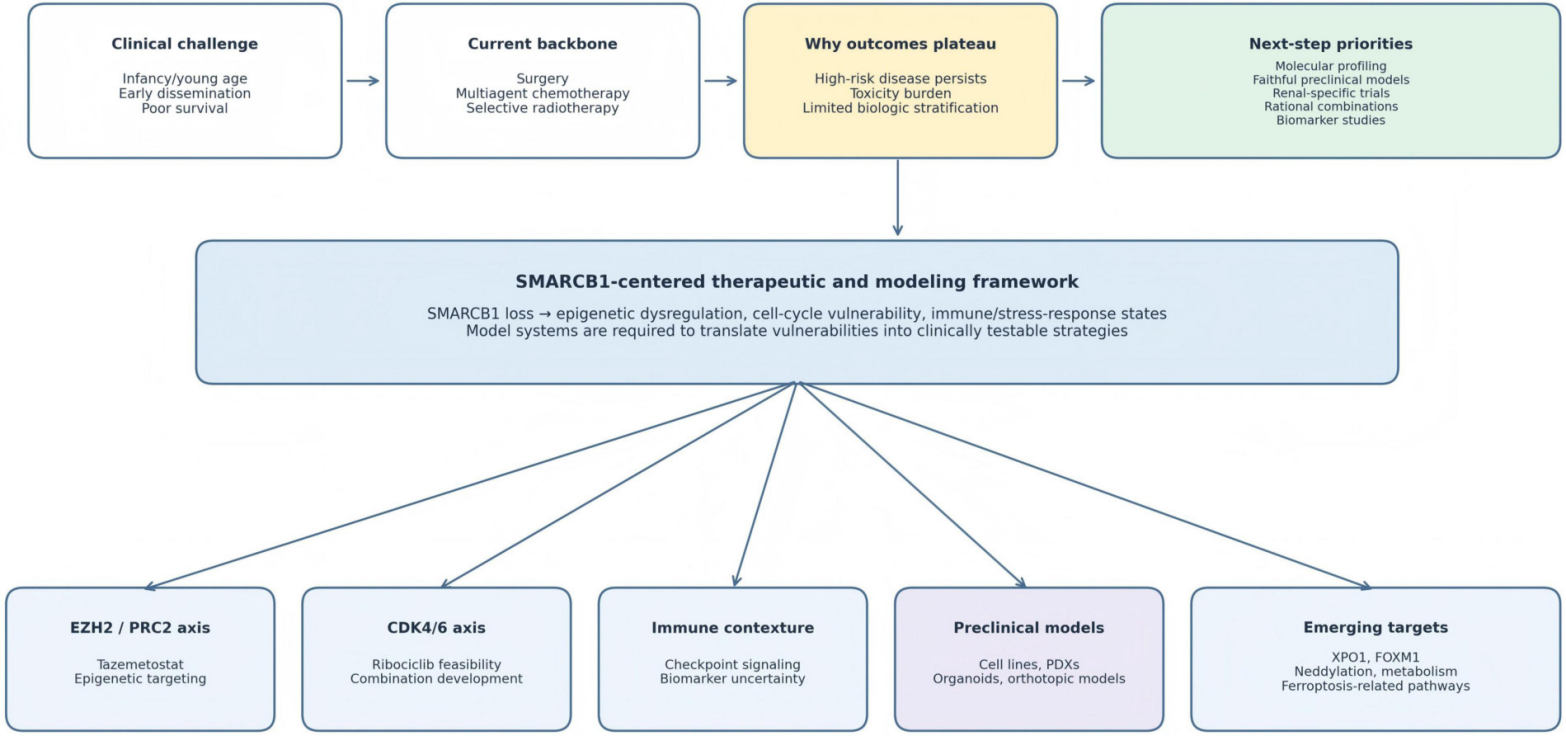
From conventional multimodal therapy to renal-specific precision care in malignant rhabdoid tumor of the kidney. The figure summarizes the current therapeutic framework of MRTK. Conventional multimodal therapy, including surgery, intensive chemotherapy, and selective radiotherapy, remains essential for immediate disease control. However, outcomes remain poor in infants and in patients with stage III/IV or metastatic disease, highlighting the limitations of further empiric treatment escalation. Loss of SMARCB1 provides the biologic basis for emerging precision strategies, including EZH2 inhibition, CDK4/6 blockade, and other biomarker-informed approaches. The next major advance is likely to depend on molecular profiling at diagnosis, faithful preclinical model systems, renal-specific collaborative trial design, rational combination strategies, and correlative biomarker studies.

Consequently, an important translational priority is not merely to add molecular profiling, but to couple profiling with systematic model generation. Future MRTK programs should prospectively establish paired tumor-normal organoids, PDXs, and, when feasible, orthotopic or metastatic models; annotate them with SMARCB1 alteration type, germline status, tissue of origin, treatment exposure, metastatic behavior, and drug response; and share them through collaborative rare-tumor networks. Such infrastructure would make it possible to test rational combinations, identify mechanisms of resistance, and determine which vulnerabilities are robust across models rather than artifacts of a single experimental system. The overall therapeutic framework and future translational roadmap are summarized in Figure 1. Key current treatment strategies and translational opportunities in MRTK are summarized in Table 1.

**Table 1:** Current treatment strategies and translational opportunities in malignant rhabdoid tumor of the kidney.

Strategy	Rationale	Current evidence	Main limitation	Near-term implication
Conventional multimodal therapy	Surgery, intensive chemotherapy, and selective radiotherapy remain essential for immediate disease control and local management.	Backbone of current care; AREN0321 improved outcomes mainly in stage I/II disease.	Advanced-stage and infant outcomes remain poor; toxicity is substantial.	Still standard; should be integrated with molecular stratification rather than abandoned.
EZH2 inhibition	SMARCB1 loss creates PRC2/EZH2 dependence and epigenetic repression.	Tazemetostat showed prolonged disease stabilization in a subset of SMARCB1/SMARCA4-altered pediatric tumors.	Single-agent activity is modest; renal-specific data are limited.	Most suitable as a component of biomarker-driven trials and rational combinations.
CDK4/6 inhibition	SMARCB1 loss perturbs cell-cycle control and may create vulnerability in the cyclin D-CDK4/6-RB axis.	Pediatric phase I ribociclib trial established feasibility but limited responses.	Predictive biomarkers are immature; optimal partners remain undefined.	Promising for combination development rather than standalone use.
Immunotherapy	Some SMARCB1-deficient tumors display immune infiltration and checkpoint target expression.	Evidence in MRTK is extrapolated mostly from mixed SMARCB1-deficient cohorts and case-level reports.	Renal-specific biomarkers, timing, and patient selection remain unclear.	Investigational; best pursued in correlative, combination-based studies.
Preclinical model systems and emerging strategies	Cell lines, PDXs, organoids, and genetic perturbation systems support mechanistic testing of SMARCB1-deficient biology and candidate vulnerabilities.	MRTK-relevant cell lines, PDXs, pediatric kidney tumor organoid biobanks, and MRT organoid screens have identified vulnerabilities such as XPO1 inhibition, neddylation, nucleotide synthesis, FOXM1 signaling, and ferroptosis-related pathways.	Models remain limited in number, often non-renal-specific, and incompletely capture immune, stromal, vascular, metastatic, and treatment-exposure contexts.	Model generation should be integrated prospectively into collaborative trials and biomarker studies.

CDK4/6, cyclin-dependent kinases 4 and 6; EZH2, enhancer of zeste homolog 2; MRT, malignant rhabdoid tumor; MRTK, malignant rhabdoid tumor of the kidney; PDX, patient-derived xenograft; SMARCB1, SWI/SNF-related matrix-associated actin-dependent regulator of chromatin subfamily B member 1; XPO1, exportin 1.

## 7 Discussion

MRTK remains one of the clearest examples in pediatric oncology of a disease in which conventional therapy is necessary but not sufficient. Surgery, intensive chemotherapy, and carefully selected radiotherapy continue to provide the foundation for treatment, particularly because many patients present with rapidly progressive disease requiring immediate control. Yet the available evidence also indicates that further nonspecific intensification alone is unlikely to deliver major survival gains, especially for infants and for patients with stage III/IV or metastatic tumors.

The most important conceptual advance in recent years has been the repositioning of MRTK as a SMARCB1-driven epigenetic malignancy rather than simply a rare high-risk pediatric renal tumor. This shift has practical therapeutic consequences. It redirects attention from empiric escalation toward molecularly anchored vulnerabilities involving chromatin regulation, cell-cycle control, apoptotic priming, and adaptive stress pathways. At present, however, precision approaches should be viewed as additive and investigational rather than replacement-based. The available clinical data support biologic plausibility and early proof of concept, but they do not yet justify displacement of conventional multimodal therapy.

Accordingly, the next major advance in MRTK is unlikely to come from a single new drug alone. Progress will more likely depend on an integrated development strategy that links molecular profiling, pathology-based diagnosis, faithful experimental models, rational combinations, renal-specific subgroup analysis, modern rare-cancer trial frameworks, and correlative biomarker science from the time of diagnosis (25–41). For a rare and lethal pediatric cancer with limited therapeutic margin, this integrated framework is not merely desirable; it is necessary. Future studies should therefore move beyond descriptive precision oncology and determine whether biomarker-informed, model-guided therapeutic strategies can improve survival, reduce treatment-related toxicity, and ultimately establish a new standard of care for children with MRTK.
